# Effect of fluoride treatment on gene expression in tea plant (*Camellia sinensis*)

**DOI:** 10.1038/s41598-017-08587-6

**Published:** 2017-08-29

**Authors:** Qing-Sheng Li, Xiao-Ming Lin, Ru-Ying Qiao, Xin-Qiang Zheng, Jian-Liang Lu, Jian-Hui Ye, Yue-Rong Liang

**Affiliations:** Tea Research Institute, Zhejiang University, Hangzhou, 310058 China

## Abstract

Tea plant is a typical fluorine (F) accumulator. F concentration in mature tea leaves is several hundred times higher than that in normal field crops. Long-term consumption of teas with high level F will increase the risks of dental and skeletal fluorosis. The mechanism of F accumulation in tea stands unclear. RNA-Seq and digital gene expression (DGE) techniques were used to investigate the effect of F on the differential expressions of transcriptome in tea plant. The results showed that F content in mature tea leaves was increased with increase in F concentration of cultural solution and duration of F treatment time. Based on comparison with data of GO, COG, KEGG and Nr databases, 144 differentially expressed unigenes with definite annotation were identified. Real-time reverse transcription PCR (qRT-PCR) was used to validate the effect of F on expression of 5 unigenes screened from the 144 unigenes. F treatment induced the expression of defense genes such as receptor-like kinases (RLKs) and U-box domain-containing protein. Based on the present study, F uptake is considered to be related to calcium-transporting ATPase, especially autoinhibited Ca^2+^ ATPase (ACAs) which was activated by the RLKs and worked as a carrier in uptake of F by tea plant.

## Introduction

Tea plant is known as a fluorine (F) accumulating plant. Mature tea leaves have much higher F concentration than normal field crops such as rice and wheat^1^. Some brick teas prepared using mature tea shoots had F content more than 800 mg/kg^2^. Long-term consumption of brick teas containing high level F would lead to excessive intake of F and increase the risks of dental and skeletal fluorosis^3^. For many higher plants, F uptake is a passive process through extra-cellular pathways by leaking past the endodermal barrier at the root tips and the uptake is linearly correlated with environmental F concentration^4^. F is not an essential element to plant and high-level F is phytotoxic to many higher plants because it inhibits respiration and photosynthesis^5^. Hydroponic experiments showed that many factors affected the F uptake by plant, such as plant species, F concentration, pH of culture medium, duration of F exposure, and interaction between fluoride and other environmental mineral elements such as Al^3+^ and Ca^2+^ ^4, 6^. It was reported that chlorine competitively inhibited F transfer in plant and aquaporins might be involved in the transmembrane transport of F^7^.

Tea plant enriches a large amount of fluoride in mature leaves without toxicity symptoms. The F accumulation mechanism in tea plant remains to be investigated. Revealing the molecular mechanism of F accumulation in tea plant will be helpful to develop agronomic techniques to controlling F accumulation in tea leaves and to develop screening indicators for breeding new tea cultivars with decreased level of leaf fluoride.

Ribonucleic acid sequencing (RNA-Seq) is a recently developed approach to transcriptome profiling, which provides a more precise measurement of levels of transcripts and their isoforms than previous methods^8^. Digital gene expression (DGE) follows the RNA-Seq protocol and it can effectively determine the differential expression of genes under experimental conditions^9^. These high-throughput sequencing and high-performance computing technology are comprehensive and rapid methods for measuring expression levels of various genes in specific tissues at a particular state of an organism. Indeed, DGE can reveal novel genes related to development and pathogenicity. Compared to microarray, DGE is a more powerful tool for searching differentially expressed and low-abundance transcripts. In the present study, we used DGE method to search differentially expressed genes under various F levels. Quantitative real-time RT-PCR (qRT-PCR) was used to verify the relationship of expression levels of the DGE screened genes to F treatment in tea plant.

## Results

### Effect of cultural solution F concentration on F accumulation in tea leaves

**Figure 1:**
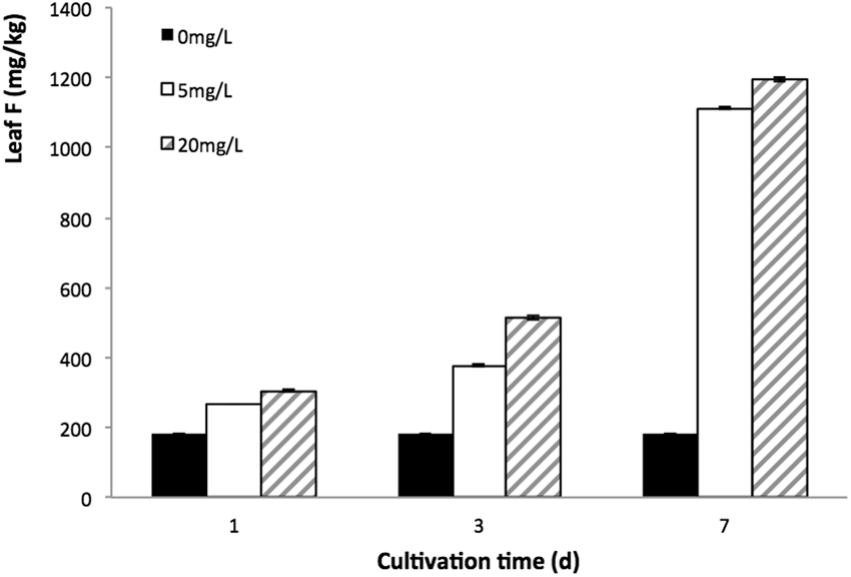
Effect of fluoride treatment on F level in tea leave.

During the testing period, tea leaf F concentration in the control group without F addition remained at a same level. However, F concentration in both 5 mg/L and 20 mg/L F treatments increased with the increase in F level of culture solution and F exposure time (Fig. 1). On the 7th day of F treatment, F concentrations in the both F treatments were more than six times of that of control, suggesting environmental F level and F exposure time have great impact on tea leaf F concentration.

### RNA-Seq, DGE and de novo assembly

**Figure 2:**
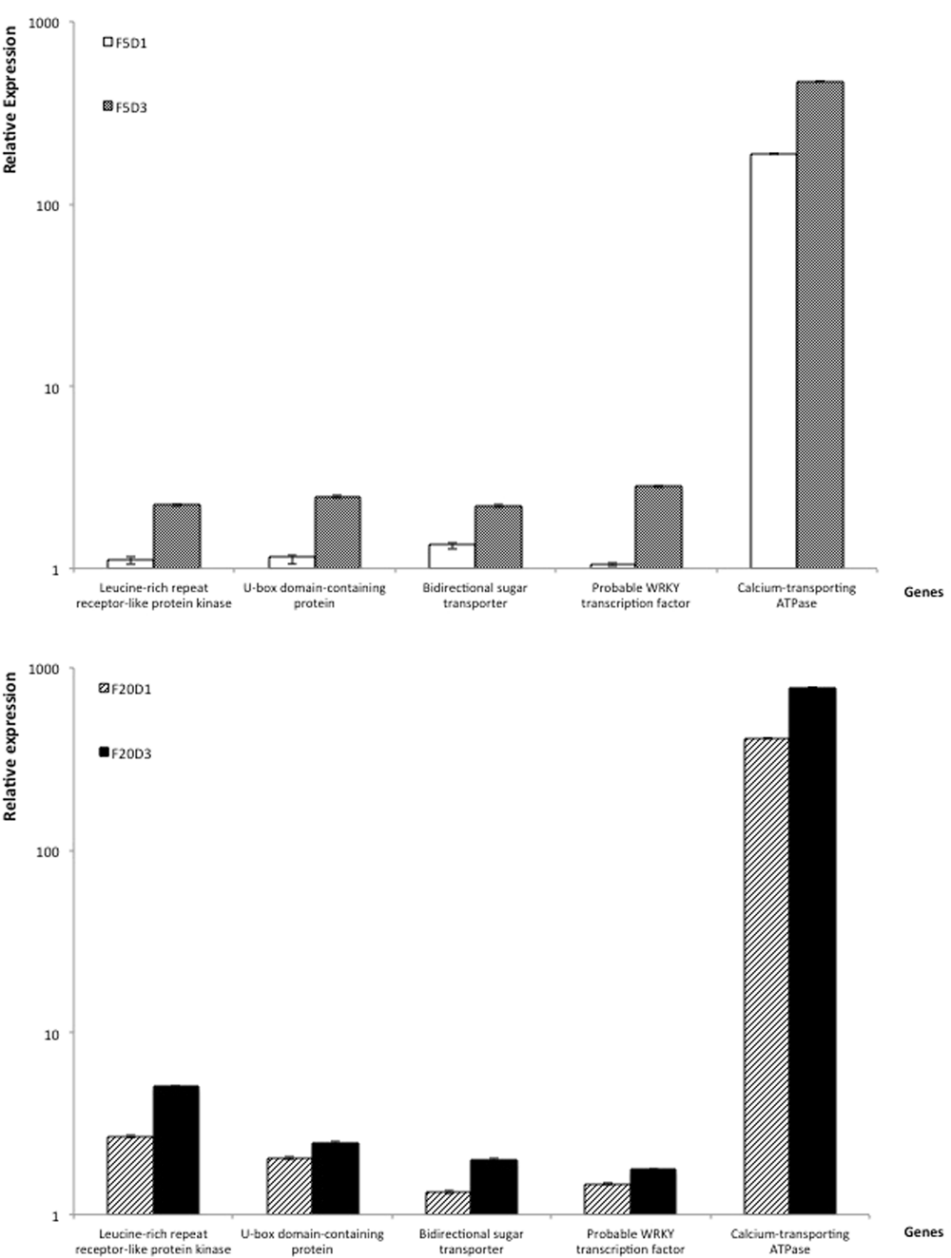
Differentially expressed genes with F dose-dependent and exposure time-dependent. F5D1: 5 mg/L F for 1 day; F5D3: 5 mg/L F for 3 days; F20D1: 20 mg/L F for 1 day; F20D3: 20 mg/L F for 3 days.

Trinity, a single *k-mer* assembler, is a good tool to *de novo* assemble short-read RNA-Seq raw data^10^. A transcriptome was obtained by Trinity using mixed tea leaf sample. The transcriptome was approximately 66 giga base pairs (Gbp), 219 thousand contigs with max length 7149 bp, and 117 thousand transcripts with 45.01% GC and 95% Q30. When the low-quality nucleotides were trimmed by Trinity, 84913 unigenes were obtained with N50 of 771 bp, in which 43619 unigenes were given functional annotations and 11774 unigenes were more than 1000 bp in length. The sequence assembly of the tested samples showed that total reads were more than 27 million, with average 53% of GC and 95% of Q30 (Table 1). The unigenes with expression strength being 2-fold higher or 50% lower than the control were defined as differentially expressed unigenes. The number of differentially expressed unigenes was 4805 in all the tested groups, among which 193 markedly differentiated unigenes were screened and 144 of them were given annotations. Further comparisons revealed that the expressions strength of five unigenes out of the 144 annotated unigenes were both F dose-dependent and F exposure time-dependent (Fig. 2). The related information of the 5 genes corresponding to the 5 unigenes was obtained by BLAST search in database of National Center for Biotechnology Information (Table 2). qRT-PCR was used subsequently further to verify the expression patterns of these five genes.

**Table 1:** Summary of the sequence assembly for each treatment group.

Samples	Total reads	Total nucleotides (bp)	GC%	Q30%
Control-1	22,498,116	3,374,717,400	52.59	95.34
Control-2	28,105,672	4,215,850,800	52.68	95.38
F5D1-1	23,992,856	3,598,928,400	52.85	95.4
F5D1-2	31,759,734	4,763,960,100	53	95.34
F5D3-1	25,744,530	3,861,679,500	52.23	95.3
F5D3-2	27,129,926	4,069,488,900	52.37	95.36
F20D1-1	25,418,168	3,812,725,200	52.65	95.33
F20D1-2	31,876,778	4,781,516,700	53.36	95.34
F20D3-1	26,754,336	4,013,150,400	52.75	95.25
F20D3-2	27,129,926	4,093,988,400	52.86	95.3
Average	27,041,004	4,058,600,580	53	95

Control: sample collected before F treatment; F5D1: 5 mg/L F for 1 day; F5D3: 5 mg/L F for 3 days; F20D1: 20 mg/L F for 1 day; F20D3: 20 mg/L F for 3 days; 1 and 2 for biological replicates.

**Table 2:** The information of 5 selected unigenes from DGE validated by qRT-PCR.

Unigene ID	Gene ID	Identity	Gene symbol	Description
c46934_g3	7455276	64%	POPTR_0019s10720 g	Leucine-rich repeat protein kinase
c9240_g1	110410305	79%	LOC110410305	U-box domain-containing protein
c42787_g1	105797095	81%	LOC105797095	Bidirectional sugar transporter
c41019_g2	100256306	55%	LOC100256306	Probable WRKY transcription factor
c10652_g1	108203676	50%	LOC108203676	Calcium-transporting ATPase

### Annotation and classification of unigenes

**Figure 3:**
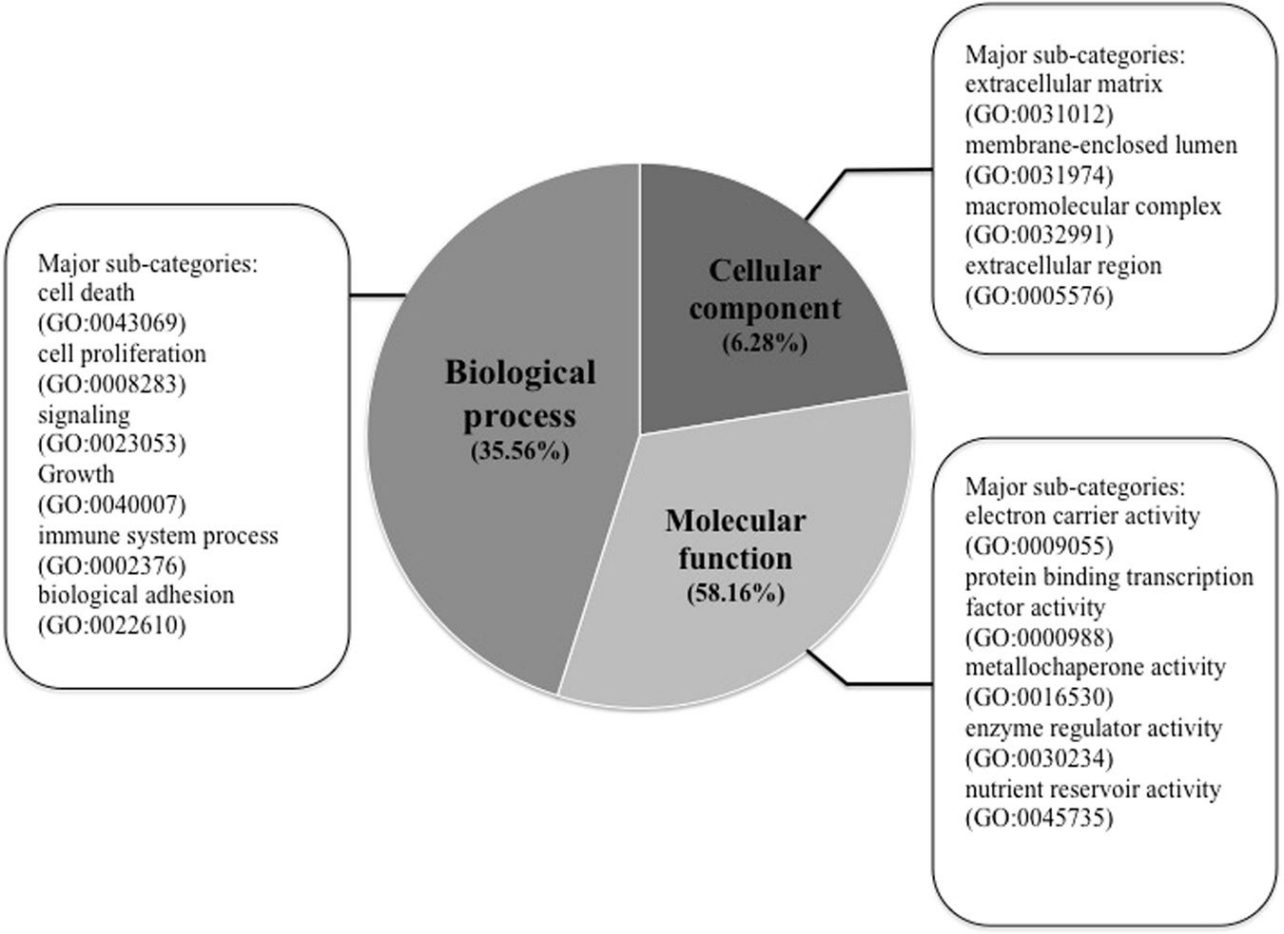
Major sub-categories of GO annotations.

Gene Ontology (GO) is a dynamic vocabulary comprehending the accumulation and variation of gene or protein functions in cell that can be applied to eukaryotes, in which there are three independent ontologies, i.e., molecular function, biological process and cellular component^11^. To analyze the function of the assembled transcripts, non-redundant sequences were submitted to a BLASTx (E-value ≤ 10^−5^) for searching against GO database, and major annotations of the differentially expressed unigenes were shown in Fig. 3. To functionally categorize the assembled transcripts, GO terms were assigned to each transcript based on the best BLASTx hit from the nr database. 239 annotations were given to the above 144 F-induced differentially expressed unigenes, among which 85 (35.56%) were related to biological processes, 139 (58.16%) to molecular functions and 15 (6.28%) to cellular components. Based on these annotations, it is suggested that F had greater impact on expression of genes involving in molecular function and biological process than the genes involving in cellular component. The results also revealed that genes involving in cell death and immune system processes were differentially expressed by F exposure, suggesting F imparted stress on the tested tea plants. It was reported that tea plant uptook F *via* active and passive pathways^4^. The differential expression of genes involving electron carrier activity and metallochaperone activity might play a role in the active F absorption process.

The database of Clusters of Orthologous Groups of proteins (COGs) is a phylogenetic classification of the proteins encoded in completely sequenced genomes, which is suitable for functional and phylogenetic annotation of newly sequenced genomes^12^. Among the above 144 F-induced differentially expressed unigenes, 136 were assigned to the appropriate COG clusters, involving in “signal transduction mechanisms”, “defense mechanisms”, “protein turnover”, “chaperones”, “secondary metabolites biosynthesis”, “transport and catabolism”, “cell wall/membrane/envelope biogenesis”, “inorganic ion transport and metabolism”, and “lipid transport and metabolism”, suggesting the F treatment affected the genes relating to signal transduction, defense mechanisms, protein turnover and chaperones. The alteration of genes involving cell wall/membrane/envelope, lipid transport and metabolism implies that tea plant might uptake F through both intracellular pathway and extra-cellular pathway by leaking through endodermal barrier.

### Validation of the differentially expressed genes by qRT-PCR

**Figure 4:**
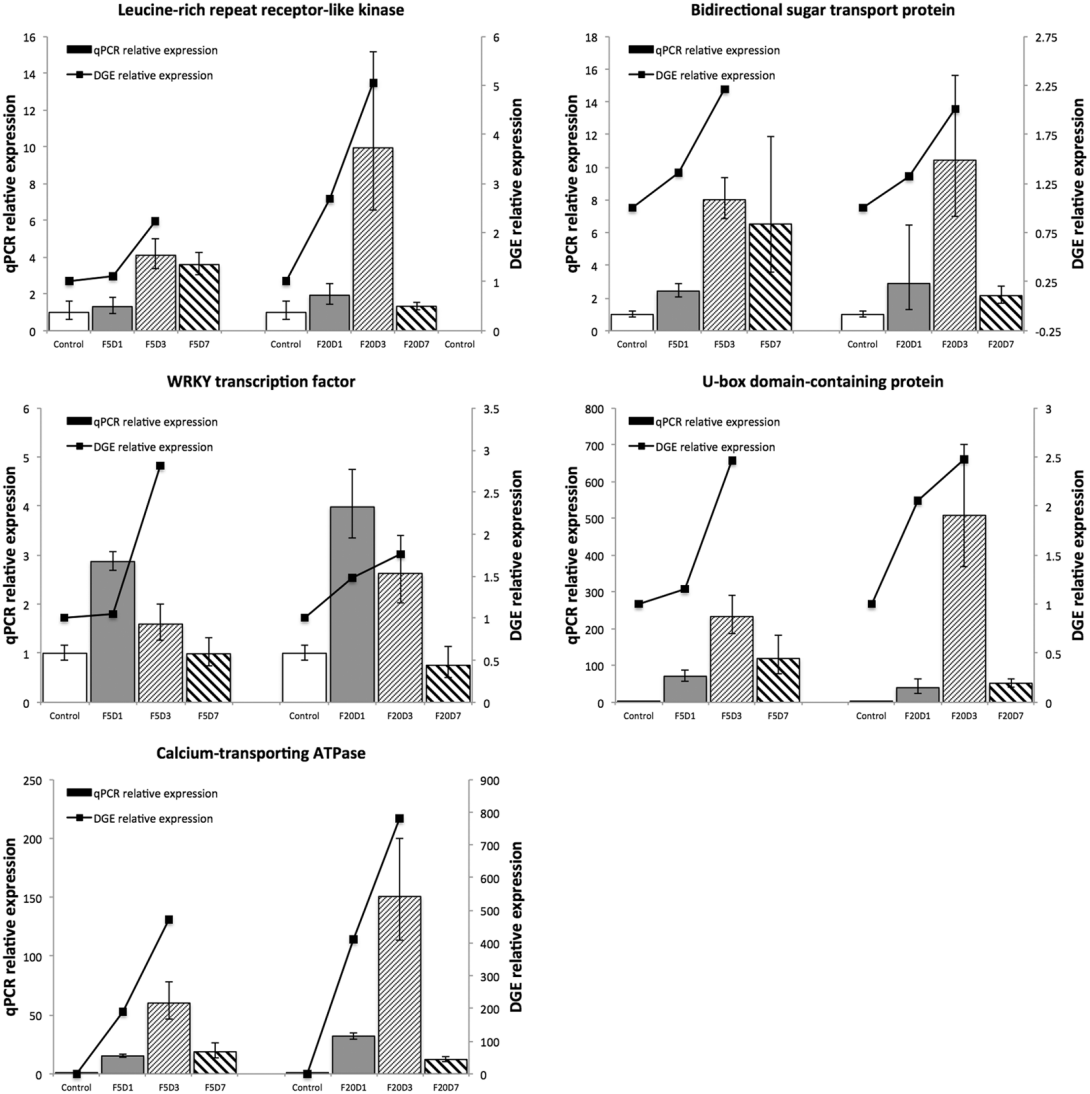
Effect of Fluoride treatment on expression of screened genes.

Though DGE is widely utilized in transcriptome study, it is limited to measure the abundance of transcripts because several transcripts might share a same tag as two unrelated genes, paralogs or alternatively spliced isoforms are involved^13^. The qRT-PCR method is usually used to further verify the DGE results. The verification results of the above five unigenes were listed in Table 2 and it showed that the highest expression strength was observed on third day after F treatment except for that of unigene c41019_g2 (Gene symbol: LOC100256306, Gene ID: 100256306) whose expression was the highest on the first day of F treatment (Fig. 4). On the third day of F treatment, the expression of all the 5 tested unigenes showed dose-dependent effect, which increased with the increase in F concentration. On the 7th day, high-level F treatment (F20D7) had weaker expression than the low level F treatment (F5D7) (Fig. 4). Unigenes c9240_g1 (Gene symbol: LOC110410305, Gene ID: 110410305) and c10652_g1 (Gene symbol: LOC108203676, Gene ID: 108203676) were very weakly expressed in the control without F addition. However, low-level F (5 mg/L) strongly stimulated their expression on the third day of F treatment (F5D3). These results suggest that the optimum sampling time in F-induced test was third day of F-exposure. The decrease in expression strength in treatments of the high level F (20 mg/L) on the seventh day (F20D7) might be a toxic effect of the F stress.

## Discussion

Fluoride treatment triggers defense related genes differentially expressed in tea plant. Receptor-like protein kinases (RLKs) are a superfamily of signal transduction genes encoded by plant genomes, which perceive different signals from the distal cells responding to stresses and diseases^14, 15^. The RLKs gene superfamily has more than 600 members, which can be divided into 44 sub-families in *Arabidopsis* ^16^. The present study revealed that there were 740 unigenes which were given functional annotations relating to RLKs. 134 out of the 740 members were involved in leucine-rich repeat protein kinase such as unigene c46934_g3 (Gene symbol: POPTR_0019s10720g, Gene ID: 7455276), 40 involved in cysteine-rich receptor like protein kinase, and 5 involved in receptor-like protein kinase *haiku2*. Leucine-rich repeat protein kinase is the largest group of RLKs involving in defense, while the cysteine-rich receptor like protein kinase plays a role in oxidative responses of these receptors in extracellular domains. The gene *haiku2* is a mutant allele of gene *iku2*, which is a leucine-rich repeat kinase gene involving in regulation of seed size^17^. The qRT-PCR in this study revealed that F showed stimulative effect on expression of RLK gene POPTR_0019s10720g in a dose-dependent manner on third day of F treatment. This can be considered to be a signaling response of tea plant to F treatment. The decrease in the expression of POPTR_0019s10720g on seventh day of F treatment might be a symptom of F stress.

U-box domain-containing proteins (U-box proteins) are a group of ubiquitin-protein ligase E3 that determines the substrate specificity in ubiquitin-26s proteasome pathway (UPP). In *Arabidopsis*, there are 37 predicted proteins containing U-box, which are classified into 5 subclasses based on structural characteristics and phylogenetic tree^18^. Though the specific functions of U-box proteins remain to be further investigated, they are largely considered to be involved in biotic and abiotic stress responses^19^. The present study revealed that there were 124 unigenes involved in U-box proteins whose expression was induced by F treatment, such as unigene c9240_g1 (Gene symbol: LOC110410305, Gene ID: 110410305) (Fig. 4). The LOC110410305 was up-regulated on the third day of F treatment, but was down-regulated on the seventh day of F treatment in F dose-dependent manner. The responses of U-box proteins to F induction on the seventh day of F treatment are considered to be a symptom of F-induced injury of tea plant.

**Figure 5:**
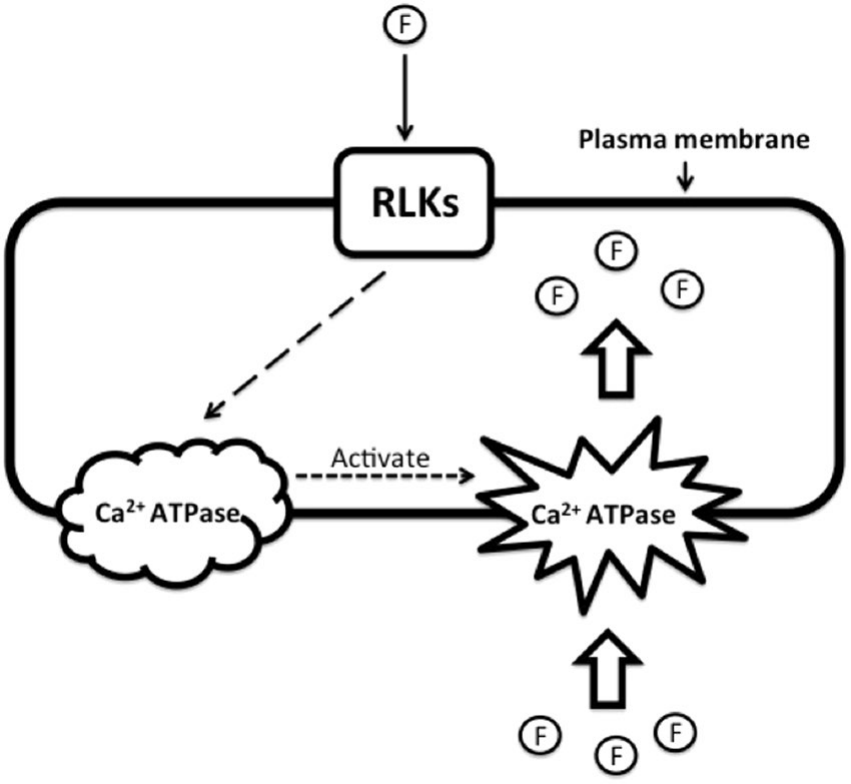
Hypothetical schematic diagram for fluorine uptake. The RLKs activate Ca^2+^ ATPase after perceiving F signals, and Ca^2+^ ATPase helps F enter into cells.

Fluoride uptake in tea plant is closely related to plasma membrane, especially Ca^2+^ pumps and channels. F uptake by tea root is partially considered to be an active and energy-dependent process involved in transmembrane^20^. The present study revealed that there were 77 genes related to calcium-transporting ATPase, in which 61 were related to calcium-transporting ATPase as autoinhibited Ca^2+^ ATPase (ACAs), including 21 ACA9, 6 ACA2, 6 ACA13, 5 ACA1 and others. Ca^2+^ triggers numerous cell biological processes such as modulating protein kinases, ion channels, and other cellular control proteins. These pathways are involved in plasma membrane-associated and intracellular control channels. The plasma membrane Ca^2+^ channels include voltage-dependent permeable Ca^2+^ channel, abscisic acid-activated Ca^2+^-permeable channel and stretch-activated channel, while the intracellular organelles Ca^2+^ channel includes insP3-activated Ca^2+^ channel, cADPR-activated Ca^2+^ release channel, Ca^2+^-induced Ca^2+^ release channel and voltage-dependent Ca^2+^ channel^21^. The ACAs in *Arabidopsis thaliana* can be divided into 4 clusters, based on sequence alignments and intron numbers and/or position^22^. Isoforms from clusters 1, 2 and 4 have been characterized, in which ACA2 plays a role in relieving salt hypersensitivity^23^. However, ACA12 and ACA13, as members of cluster 3, are barely investigated. Unlike other ACAs, ACA12 is not stimulated by calmodulin. ACA12 and ACA13 are highly expressed under stress conditions^24^. The LOC108203676 equivalent to ACA9 in the present study was dramatically upgraded by F induction on first day (Fig. 4), suggesting ACA9 might be involved in responses of F uptake or F stress in tea plant. It is hypothesized that the environmental F triggers the expression of RLKs, which in turn activate Ca^2+^ ATPase^25^. The activated Ca^2+^ ATPase might work as a carrier of fluorine in the F sorption process (Fig. 5).

F stimulation accelerates the expression of defense genes such as RLKs and U-box proteins. The RLKs is considered to play important role in the absorption of environmental fluorides. F triggers the expression of RLKs, which in turn activate Ca^2+^ ATPase. The activated Ca^2+^ ATPase promotes the absorption and transport of fluoride.

## Materials and Methods

### Fluorine treatment and leaf sampling

Two-year-old tea cuttings of *Camellia sinensis* cv. ‘JK2’ were hydroponically cultivated in an air-conditioned chamber. The plants were grown and acclimated for eight weeks in basic nutrient solution (Table 3) at 25 ± 2 °C, 70 ± 10% relative humidity and 12 h light/12 h dark. The renewal of the nutrient solution was carried out weekly. The plants were then divided into 3 groups (10 plants in each group) and treated at three levels of F^−^ (0, 5, 20 mg/L), respectively. The chemical source of F^−^ was NH_4_F, and the pH of the solution held 4.8–5.2 adjusted by 0.1 M HCl or 0.1 M NaOH. Third leaf from apical bud was sampled with three repetitions for total RNA extraction and F analysis on the day before F treatment, and 1^st^, 3^rd^ and 7^th^ day of F treatment. The samples for RNA extraction were frozen in liquid nitrogen and then stored at −80 °C till use, and those for F determination were fixed in steam for 1 min and then dried at 80 °C.

**Table 3:** Basic nutrient solution formula.

Chemicals	Concentration (mg/L)
NH_4_NO_3_	100.05
KH_2_PO_4_	34.68
K_2_HPO_4_	1.64
CaSO_4_ · 2H_2_O	2.15
MgSO_4_ · 7H_2_O	49.00
Al_2_(SO_4_)_3_ · 10H_2_O	33.32
FeSO_4_ · 7H_2_O	0.28
Na_2_SiO_3_ · 9H_2_O	14.21
H_3_BO3	5.00
MnSO4	3.00
ZnSO_4_ · 7H_2_O	0.44
CuSO_4_ · 5H_2_O	0.16
Na_2_MoO_4_ · 2H_2_O	0.16

### Determination of fluorine in tea samples

F content in the tea samples was determined using F ion selective electrode (Shanghai Ruosull Technology Co., Ltd., Shanghai China) following the method described by Stevens *et al*.^26^. The reclaim rate of the added F was 95.0–99.3%, with coefficient of variation 2.1%.

### RNA-Seq and DGE

Five representative samples were used in the RNA-Seq and DGE analysis and they included the one before F treatment (tabbed as Control) and four samples from various F level and exposure time combination treatments: 5 mg/L F for 1 day (tabbed as F5D1), 5 mg/L F for 3 days (F5D3), 20 mg/L F for 1 day (tabbed as F20D1), 20 mg/L F for 3 days (F20D3), each group has biological replicate. Total RNA was prepared using RNAprep pure Plant Kit (TIANGEN Biotech Co., Ltd., Beijing, China) according to the kit instruction. The amount of total RNA should be more than 10 μg and the concentration of total RNA should be bigger than 300 ng/μL. The mRNA was enriched using Oligo (dT) magnetic beads and fragmented in fragmentation buffer. Agilent 2100 Bioanalyzer (Agilent Technologies, Inc., Santa Clara, USA) was used to check the obtained RNA integrity, and the total RNA concentration was determined using Nanodrop 2000 (Quawell Technology, Inc., San Jose, USA). RNA-Seq and DGE were carried out following Illumina protocol^27^. The unigenes with expression strength being 2-fold higher or 50% lower than the control were defined as differentially expressed unigenes. Trinity was used to identify SNPs, small insertions or deletions and this set of transcripts was used for downstream statistical analysis. All non-redundant transcripts were searched against the GO, COG, nr database by BLASTALL package with the significant threshold of E-value ≤ 10^−5^.

### Quantitative real-time RT-PCR

Quantitative real-time RT-PCR (qRT-PCR) was used to verify the expression levels of the differentially expressed genes screened by RNA-Seq and DGE profiling. cDNA was synthesized by PrimeScript^TM^ RT reagent Kit with gDNA Eraser (TaKaRa Biotechnology Co., Ltd., Dalian, China). The qRT-PCR was carried out on the Applied Biosystems StepOnePlus Sequence Detection System (Carlsbad, CA, USA) with SYBR^®^ Premix Ex Taq^TM^ II (TaKaRa Biotechnology Co., Ltd., Dalian, China). The RNA samples used for qRT-PCR were biological replicate from the same experiment. The *β-Actin* was used as reference gene in the qRT-PCR and the test was carried out in triplicate. The relative gene expression levels were estimated by average threshold cycle which was calculated by ${2}^{-{\rm{\Delta }}{\rm{\Delta }}{C}_{T}}$ ^28^. The data were expressed as the mean ± SD (standard deviation), and the primer sequences used in the qRT-PCR were listed in Table 4.

**Table 4:** Primer sequences used for qRT-PCR reactions.

Unigene name	Sequence (5′-3′)
c46934_g3	TGAGCCAGAGTTCGGAGAGA
	TTACAAGGCTCGGCCTCTTC
c9240_g1	TCGATCGCCAACAGTTTCCA
	CGAGCATTTCGAGCAAGTCG
c42787_g1	CAAGGACCACACTACCGACC
	GAAAAGCCTGCGCAATCACA
c41019_g2	GTGGGAGGACTCGAAAGCAA
	TTCCGGTCCTGCTCAAGTTC
C10652_g1	TAGCTGATGGGTTTGCTCGG
	CTGAGGCCAGCTTGTTGAGA
